# Elimination and detoxification of 2,4-D by *Umbelopsis isabellina* with the involvement of cytochrome P450

**DOI:** 10.1007/s11356-017-0571-4

**Published:** 2017-11-14

**Authors:** Justyna Nykiel-Szymańska, Paulina Stolarek, Przemysław Bernat

**Affiliations:** Department of Industrial Microbiology and Biotechnology, Faculty of Biology and Environmental Protection, University of Lodz, Banacha Street 12/16, 90-237 Lodz, Poland

**Keywords:** Toxicity, Degradation, 2,4-D metabolites, *Artemia franciscana*, 2,4-dichlorophenoxyacetic acid

## Abstract

The chemical 2,4-dichlorophenoxyacetic acid (2,4-D) is used in agriculture as a herbicide. Its intensive use has an adverse effect on the environment. This study involved examining the degradation of 2,4-D compound by the filamentous fungus *Umbelopsis isabellina*. After 5 days of incubation, 98% of the herbicide (added at 25 mg L^−1^) was found to be removed. The elimination of 2,4-D by *U. isabellina* was connected with the formation of 2,4-dichlorophenol (2,4-DCP), which resulted in a 60% decrease in the sample toxicity toward *Artemia franciscana* larvae. The metabolism of 2,4-D was inhibited by the addition of metyrapone, a known cytochrome P450 inhibitor. It provides evidence that cytochrome P450 system is involved in 2,4-D metabolism in *U. isabellina*.

## Introduction

The auxin-like herbicide 2,4-dichlorophenoxyacetic acid (2,4-D) has been used all over the world since the 1940s (Li et al. 2017). It is an active ingredient in more than 1500 herbicide products (Aylward and Hays 2008). Pesticides not only enter straight into the target organisms but also disperse into the ecosystems, transfer to other areas, and thus pose a great danger to nontarget organisms. Therefore, the development of efficient methods for their degradation is relevant and necessary.

Bacterial degradation studies dealing with 2,4-D have been published in the literature. The herbicide-degrading strains belonging to *Achromobacter* sp., *Bradyrhizobium* sp., *Burkholderia* sp., *Cupriavidus* sp., and *Sphingobium* sp. have been reported from different parts of the world (Chang et al. 2015; Feld et al. 2015). But investigations involving fungi are rarely reported. The number of fungal strains that are active 2,4-D degraders and can be applied for the xenobiotic removal is limited. The filamentous fungus *Mortierella isabellina* (which is currently classified as *Umbelopsis isabellina*) has been mentioned as a microorganism that eliminates 2,4-D (Vroumsia et al. 1999). There is no detailed information about the mechanisms of fungal degradation of this herbicide.

In plants, the auxinic herbicide 2,4-D is known as a CYP P450 inducer (Onkawa and Inui 2015). Additionally, human cytochrome P450 3A4 is involved in the biotransformation of the herbicide (Mehmood et al. 1996). Moreover, the filamentous fungus *M. isabellina* is known for catalyzing 7a-hydroxylation of dehydroepiandrosterone as well as epiandrosterone steroid hydroxylation with cytochrome P450 monooxygenase(s) engagement (Kołek et al. 2011). These findings prompted us to extend the studies to the examination of *U. isabelina* activity during 2,4-D degradation. The present work reports the results of this pesticide metabolism by the soil fungus *U. isabellina* involving the participation of cytochrome P450.

## Materials and methods

### Reagents

2,4-D was purchased from Sigma–Aldrich (Poznan, Poland). The other chemicals were acquired from Sigma–Aldrich (Poznan, Poland) and POCh (Gliwice, Poland). All the chemicals were high-purity grade reagents. Stock solutions of 2,4-D were prepared at 5 mg mL^−1^ of ethanol.

### The strain and its growth condition

*Umbelopsis isabellina* DSM 1414 (synonym *Mortierella isabellina*), isolated from the soil of British Columbia, Canada, was purchased from the German Collection of Microorganisms and Cell Cultures (Braunschweig, Germany).

Seven-day-old spores of the *U. isabellina* strain from cultures on ZT agar slants (g L^−1^: glucose (4); Difco yeast extract (4); agar (25); and malt extract (6 °Blg), up to 1 L; pH 7.0) were used for inoculating 20 mL of a synthetic medium with 2% glucose in 100-mL Erlenmeyer flasks. The medium was composed of (g L^−1^): K_2_HPO_4_ (4.36), KH_2_PO_4_ (1.7), NH_4_Cl (2.1), MgSO_4_ × 7H_2_O (0.2), MnSO_4_ (0.05), FeSO_4_ × 7H_2_0 (0.01), CaCl_2_ × 2H_2_O (0.03), glucose (20), and distilled water (up to 1000 mL, pH = 6.5). The cultivation was performed on a rotary shaker (160 rpm) for 24 h at 28 °C. The pre-culture was transferred to a fresh medium at the ratio 1:1 and incubated for the next 24 h. The homogeneous pre-culture (10%) was introduced into the growth medium supplemented with 25 mg L^−1^ (0.11 mM) of 2,4-D or into the control culture without the tested compound. Furthermore, abiotic controls (uninoculated) were prepared. All cultures were incubated at 28 °C on a rotary shaker (160 rpm). Biomass was washed with distilled water, and dry weight was quantified by the method described by Bernat et al. (2013).

The maximum specific growth rate (*μ*_max_) was calculated using the least squares fitting to the linear part of the semilogarithmic plot of the fungal biomass versus time.

The experiments were performed in the exponential (24 h) and stationary (120 h) growth phases for control and 2,4-D-treated mycelium.

### Utilization of 2,4-D in the presence of the cytochrome P450 inhibitor

The homogeneous pre-culture (10%) prepared as presented above was transferred into 18 mL of growth medium (in 100-mL Erlenmeyer flasks) supplemented with the cytochrome P450 inhibitor metyrapone (2 mM) and 2,4-D (25 mg L^−1^). Uninoculated media with appropriate amounts of 2,4-D added in ethanol served as controls. Then, the flasks were incubated for 5 days in the same conditions (The strain and its growth condition section).

### Determination of 2,4-D and its metabolites using liquid chromatography–mass spectrometry

The herbicide and its metabolite were extracted using the Quick, Easy, Cheap, Effective, Rugged, and Safe (QuEChERS) method (Siewiera et al. 2015). Fungal cultures were transferred into 50-mL Falcon tubes. Next, 10 mL of acetonitrile with glass beads was inserted. The biomass homogenization (Retsch, Ball Mill MM 400) was performed twice for 4 min. A salt mixture (2 g of MgSO_4_, 0.5 g of NaCl, 0.5 g of C_6_H_5_NaO_7_ × 2H_2_O, and 0.25 g of C_6_H_6_Na_2_O_7_ × 1.5 H_2_O) was added to the homogenate and mixed for 2 min. Then, the sample was centrifuged for 5 min (4 °C at 2000×*g*). Finally, 3 mL of the top layer was collected for the chromatographic analysis. Next, the quantitative analyzes of the compounds were performed by using liquid chromatography–mass spectrometry (LC–MS/MS) (LC Agilent 1200 coupled with tandem mass spectrometer AB Sciex QTRAP 4500). The separation was conducted using a capillary Eclipse XDB-C18 column (50 mm × 4.6 mm, 4.6 μm) maintained at 37 °C. For sample separation, the mobile phase consisting of water (A) and methanol (B) supplemented with 5 mmol L^−1^ ammonium formate was used. The run time was 6 min with the solvent gradient initiated at 20% B. After 1 min, that is, during the next minute, B was increased to 100% and maintained at 100% for additional 4 min before returning to the initial solvent composition more than 2 min. The flow rate was 600 μL min^−1^. The detection of the pesticide was conducted using an MS/MS acquisition in the multiple reaction monitoring (MRM) negative ionization mode. The monitored MRM pairs were *m*/*z* 218.9 > 161 and 220.9 > 163 at 2.91 retention time.

The same chromatographic method was used for identifying possible 2,4-D metabolites, which could be detected in previously extracted samples, the mass spectrometric detection was conducted on a QTRAP 4500, working in the negative ionization mode. Information-dependent acquisition (IDA) methods—enhanced MS (EMS)/enhanced product ion (EPI) and precursor ion (Prec)/EPI—were used for identifying possible metabolites. The spectra were obtained over a range from *m*/*z* 50 to 600.

### Determination of 2,4-D metabolites using gas chromatography–mass spectrometry (GC/MS)

GC/MS was also used for the identification of possible degradation products. The fungal culture was homogenized with glass beads twice for 4 min at 25 m s^−1^ (Retsch, Ball Mill MM 400). Next, the samples were extracted twice with ethyl acetate. The extracts were dried with anhydrous sodium sulfate, evaporated under reduced pressure at 40 °C, and 2 mL of ethyl acetate was added.

The analysis was performed with an Agilent Model 7890 gas chromatograph, equipped with a 5975C mass detector. The separation was performed using a capillary column HP 5 MS methyl polysiloxane (30 m × 0.25 mm id × 0.25 mm ft). The column temperature was maintained at 60 °C for 3 min, then increased to 250 °C at the rate of 10 °C min^−1^ and finally to 280 °C at the rate of 20 °C min^−1^. The column temperature was maintained at 280 °C for 5 min. Helium was used as a carrier gas at a flow rate of 1 mL min^−1^. The injection port temperature was 250 °C. Split injection was used. The identification of possible metabolites was conducted using NIST MS 14 software.

### Toxicity study

The toxicity bioassay Artoxkit M (MicroBioTests, Inc., Mariakerke, Belgium) was used for checking the acute toxicity by testing the post-culture extracts of the fungus *U. isabellina*, cultivated with or without the tested compound. Changes in the toxicity of the samples were determined using the larvae of the test crustaceans *Artemia franciscana* in accordance with standard operational procedures. The fungal cultures (with and without 2,4-D for 24 and 120 h) were homogenized using a ball mill. The homogenized samples and abiotic controls were extracted twice with ethyl acetate, dried with anhydrous sulfate, and evaporated under reduced pressure at 40 °C. The extracts were dissolved in 0.1 mL of ethanol and diluted with saline water to obtain the initial volume of the cultures (20 mL). Next, appropriate dilutions were performed. *A. franciscana* controls with saline water and with the same volume of ethanol as in the test samples were also prepared. The toxicities of 2,4-D and its metabolites were calculated as a percentage of immobile larvae after 48 h of incubation. The larvae motility was measured microscopically at a magnification of 40×.

### Data acquisition and statistical analysis

The experimental data represent the means of at least three independent experiments. Sample variability is given as a standard deviation (± SD). The significance of differences between the control and the treatment mean values was determined by Student’s *t* test. Differences at *P* < 0.05 were considered significant. Statistical analyzes were performed using Excel 2013 (Microsoft Corporation, USA).

## Results

### Growth of the *Umbelopsis isabellina* strain in the presence of 2,4-D

**Fig. 1:**
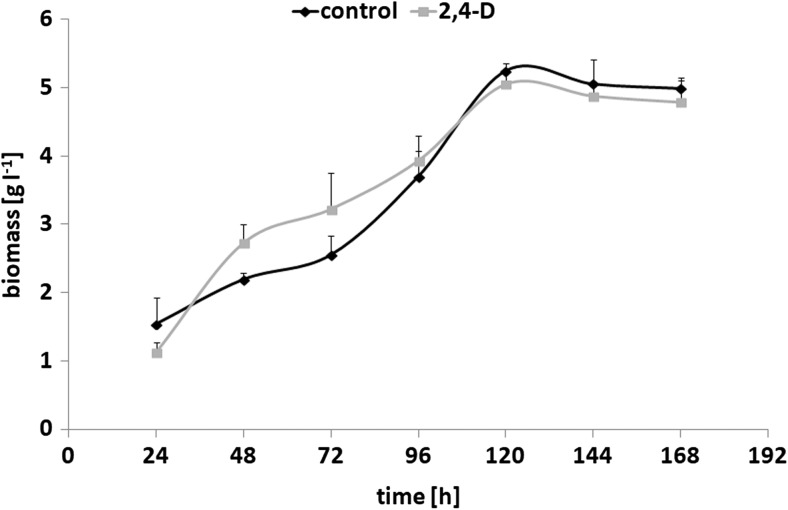
Growth curves of *U. isabellina* strain cultivated on a synthetic medium with or without 2,4-D (added at 25 mg L^−1^)

Fungal biomass growth after the exposure to 2,4-D (25 mg L^−1^, 0.11 mM) was investigated. This concentration resulted in the toxic effects of 2,4-D on zebrafish embryonic development or on *Pimephales promelas* (*Cyprinidae*) (Alexander et al. 1985; Li et al. 2017). After 5 days of incubation on the synthetic medium, the dry fungal weight for the control biomass was estimated at a concentration of 5.25 g L^−1^ (Fig. 1). In the samples with the herbicide from 120 h, a slight decrease in the fungal growth was observed. Moreover, after exposure to 2,4-D, the maximum specific growth rate (*μ*_max_) decreased from 0.015 h^−1^ to 0.01 h^−1^.

### Herbicide elimination by *U. isabellina*

**Fig. 2:**
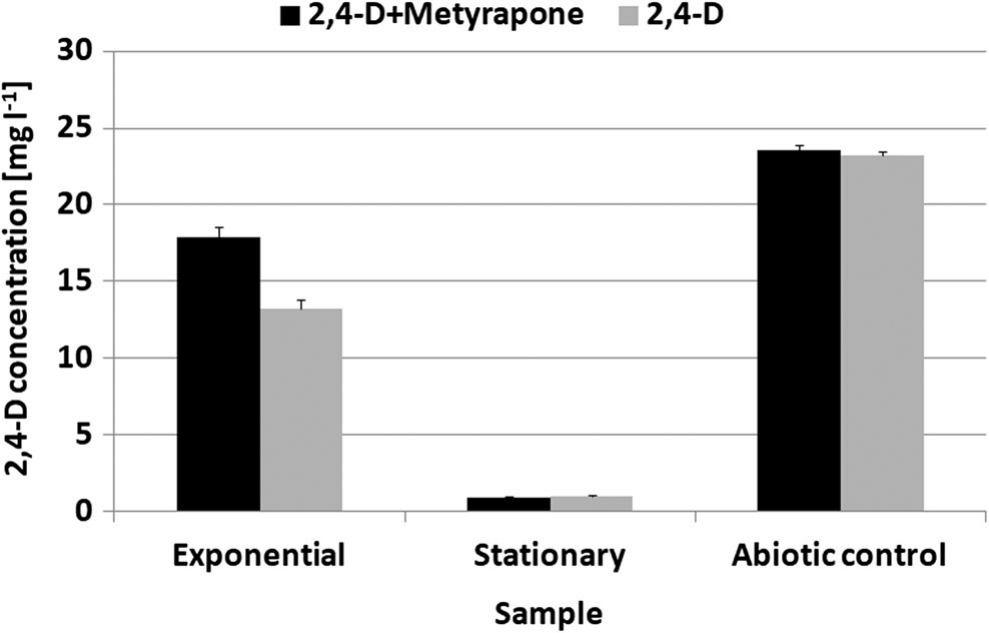
The compound 2,4-D removal by *U. isabellina* cultures incubated on a synthetic medium for 24 h (exponential phase of growth) and 120 h (stationary phase of growth) with cytochrome P450 inhibitor—metyrapone (2 mM) (2,4-D added at 25 mg L^−1^)

In the next part of the investigation, the time course of 2,4-D transformation was studied. A degradation of the xenobiotic was already observed during the initial 24 h of incubation. Moreover, the effectiveness of the pesticide removal after 120 h of culturing reached almost 94% (Fig. 2).

**Fig. 3:**
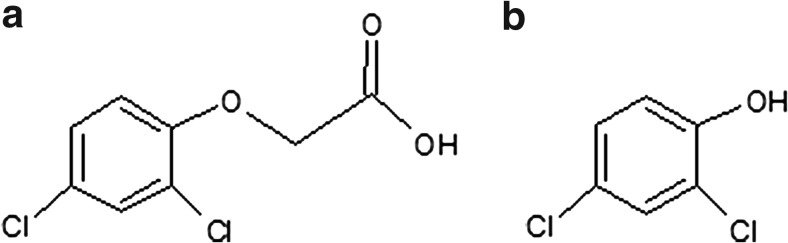
Chemical structures of the tested molecules (2,4-D (**a**), 2,4-DCP (**b**))

The 2,4-D metabolic intermediate, 2,4-dichlorophenol (2,4-DCP) (Fig. 3), was determined by using LC–MS/MS and GC/MS techniques. LC–MS/MS chromatograms revealed the presence of a possible 2,4-D metabolite with the hydroxyl group at the retention time 3.12 (*m/z* at 235, 237, 239). This metabolite was also reported in the study describing the mechanisms of 2,4-D degradation by oxalate-mediated photooxidation (Kwan and Chu 2004).

### Cytochrome P450 involvement in 2,4-D degradation by the fungus

In several plants, it has been shown that cytochrome P450 enzymes are involved in the degradation of 2,4-D (Torra et al. 2017). To find out whether cytochrome P450 is involved in the herbicide metabolism by *U. isabellina*, an experiment with metyrapone, the cytochrome P450 competitive inhibitor, was performed. Metyrapone (2 mM) did not affect the growth of the fungus. Moreover, its presence led to a significant inhibition of 2,4-D degradation reactions (*P* < 0.01). After a 24-h incubation (the exponential phase of growth) of the fungus with the inhibitor and 2,4-D, the xenobiotic removal efficiency was lower by about 18.5% (Fig. 2). At the end of culturing, the influence of metyrapone was not visible. Probably, if the inhibitor concentration had been higher, the pesticide transformation would have been less efficient. However, higher concentrations of metyrapone influenced the microorganism’s growth. Therefore, considering the results from the first 24 h of incubation, the inhibition of hydroxylation reactions in the presence of the cytochrome P450-inhibiting substance—metyrapone—points to an involvement of these enzymes in the herbicide transformation reactions.

### Toxicity assessment

Products generated as a result of the biodegradation of xenobiotics may have more harmful effects than the parent compounds (Bernat et al. 2013). Therefore, it is necessary to perform studies aimed at a toxicological characterization of biologically active substances. The saltwater shellfish *Artemia* spp*.* is very often used for evaluating the toxicity of a variety of xenobiotics, heavy metals, pharmaceuticals, wastewaters, or seawater (Libralato et al. 2010; Bernat et al. 2013; Słaba et al. 2013; Leis et al. 2014; Xu et al. 2015; Zawadzka et al. 2015; Janicki et al. 2016). To determine the toxicity changes during the biotransformation of 2,4-D by *U. isabellina*, an Artoxkit M bioassay was applied. The inhibition value of a pure 2,4-D solution (without incubation and extraction) on the nauplii motility was equal to 63 ± 9.81% (Table 1). The toxicity of the abiotic control was constant during the time course of the experiment. The inhibition values of the pesticide on the *A. franciscana* motility reached 61 ± 9.8 and 59 ± 8.54% for abiotic controls from 24 and 120 h, respectively. No toxicity of biotic controls (without 2,4-D) was observed in the tested aquatic invertebrates. A comparison of the mortality of *A. franciscana* for the abiotic controls and the xenobiotic-treated fungal samples after 24 h of incubation showed a threefold reduction in toxicity. Moreover, during the next 96 h of culturing, a decreasing trend in toxicity (from about 18.5 to 0%) was observed, which was associated with the 2,4-D degradation process. The test results indicated that the *U. isabellina* strain can detoxify 2,4-D.

**Table 1:** Toxicity analysis of *U. isabellina* culture with 25 mg L^−1^ 2,4-D in *A. franciscana* assay

Type of post-culture extracts	Mortality of *A. franciscana* (%)	
	24 h	120 h
Abiotic control	61 ± 9.8	59 ± 8.54
*U. isabellina*	0	0
*U. isabellina* + 2,4-D	18.5 ± 6	0
Pure 2,4-D solution (without incubation and extraction)	63 ± 9.81	

## Discussion

A slight inhibitory effect of 2,4-D on the phases of *U. isabellina* growth was observed. Moreover, an intensive herbicide degradation took place during the first 24 h of culturing. In the studies of Vroumsia et al. (2005) with 100 mg L^−1^ of 2,4-D in the Galzy and Slonimski (GS) synthetic liquid medium with *M. isabellina*, a rapid removal rate of the xenobiotic was observed after 24 h of incubation. Probably, the higher initial concentration of the herbicide influenced the fungal growth and degradation rate. In many studies, the concentration of the toxicant affected the lag phase before the onset of the degradation (Vroumsia et al. 2005). This trend was also observed in tributyltin (TBT) biodegradation by *Cunninghamella elegans*. The highest applied concentration of TBT almost completely inhibited the fungal growth and decreased the efficiency of TBT degradation (Bernat and Długoński 2009).

For several fungal strains of *Zygomycete* from the genera of *Cunninghamella*, *Mucor*, or *Umbelopsis* have been shown that cytochrome P450 enzymes are involved in the transformation of chemicals such as tributyltin, pentachlorophenol, or steroid hormones (Bernat et al. 2013; Carvalho et al. 2011; Kołek et al. 2011). The obtained results suggest cytochrome P450 involvement in 2,4-D degradation by the investigated strain of *U. isabellina*. There is evidence showing that 2,4-D induces P450 activity in rice and corn poppy (Hirose et al. 2007; Torra et al. 2017). But potential 2,4-D metabolites have not been determined. However, the metabolism of 2,4-D with the involvement of cytochrome P450 was studied using microsomal fractions and whole cells of *Saccharomyces cerevisiae* expressing cytochrome P450 3A4. The compound 2,4-DCP was identified as the only product of metabolism by thin layer chromatography followed by nuclear magnetic resonance and infrared spectroscopy (Mehmood et al. 1996). Until now, most of the reports describing microbial degradation of 2,4-D with the involvement of particular enzymes have concerned bacterial activity. *Cupriavidus gilardii* T-1 degraded 2,4-D to 2,4-DCP by a cleavage of the ether bond and then into 3,5-dichlorocatechol via hydroxylation, followed by ortho-cleavage to form cis-2-dichlorodiene lactone (Wu et al. 2017). The compound 2,4-D was degraded by the following fungal strains: *Cunninghamella elegans*, *C. echinulata*, *Rhizoctonia solani*, *Verticillium lecanii*, *Penicillium chrysogenum*, *Aspergillus penicillioides*, *Eupenicillium* sp*.*, and *U. isabellina* (Vroumsia et al. 1999; Vroumsia et al. 2005; Ferreira - Guedes et al. 2012). The compound 2,4-DCP was identified as the main product of fungal metabolism of 2,4-D. 2,4-DCP degradation by an endemic soil fungus *Mortierella* spp. was described by Nakagawa et al. (2006). The researchers identified four aromatic metabolites (chlorohydroquinone, 3,5-dichlorocatechol, 3,5-dichloroguaiacol, 4,6-dichloroguaiacol, and hydroquinone) as well as two 2,4-DCP degradation pathways (by hydroxylation and dechlorination).

Despite the high efficiency of 2,4-D in crop protection, its excessive use causes serious ecological effects. It adversely affects the biological treatment system and also causes toxicity in receiving waters (Gonzalez et al. 2012). A number of studies have been devoted to 2,4-D toxicity. The lethal doses and concentrations of the herbicide differ between species. Previous toxicity studies of 2,4-D have provided the LD_50_ values on various animal models, including *Daphnia magna* (> 100 mg L^−1^), *Artemia salina* (16.63 mg L^−1^), *Pimephales promelas* (25 mg L ^−1^), *Oreochromis niloticus* (86.9 mg L^−1^), *Oncorhynchus mykiss* (1.1 mg L^−1^), *Danio rerio* (46.71 mg L^−1^), rabbit (> 1600 mg kg^−1^), or rat (639 mg kg^−1^) (Alexander et al. 1985; Morshed et al. 2005; Sarikaya and Selvi 2005; Heggstrom 2009; Li et al. 2017). Thus far, little attention has been paid to research on the changes in toxicity during the microbial degradation of 2,4-D. Furthermore, to the best of our knowledge, there is a lack of information about the use of the Artoxkit M test in this context. Only for bacterial strain *Delftia* spp., capable of effectively eliminating 100 and 200 mg L^−1^ of 2,4-D, xenobiotic detoxification was evaluated. To confirm the absence of potentially toxic byproducts, toxicity tests were conducted using *Lactuca sativa* seeds. Toxicity was assessed by measuring the reduction in root elongation of *L. sativa*, according to EPA/600/3-88 (1989) (Gonzalez et al. 2012).

## Conclusions

The present study demonstrates that the applied pesticide was eliminated by the *U. isabellina* strain. These results confirm that the enzyme responsible for 2,4-D degradation by this fungus includes cytochrome P450 monooxygenase(s). Artoxkit M showed a significant reduction in the sample toxicity as a result of pesticide biodegradation by the filamentous fungus. The obtained results are a very good starting point for further investigation on 2,4-D-polluted soil bioremediation with *U. isabellina* strain.

## References

[CR1] Alexander HC, Gersich FM, Mayes MA (1985). Acutetoxicity of four phenoxy herbicides to aquatic organisms. Bull Environ Contam Toxicol.

[CR2] Aylward LL, Hays SM (2008). Biomonitoring Equivalents (BE) dossier for 2,4 dichlorophenoxyacetic acid (2,4-D) (CAS no. 94-75-7). Regul Toxicol Pharmacol.

[CR3] Bernat P, Długoński J (2009). Isolation of *Streptomyces sp*. strain capable of butyltin compounds degradation with high efficiency. J Hazard Mater.

[CR4] Bernat P, Szewczyk R, Krupiński M, Długoński J (2013). Butyltins degradation by *Cunninghamella elegans* and *Cochliobolus lunatus* co-culture. J Hazard Mater.

[CR5] Carvalho MB, Tavares S, Medeiros J, Núñez O, Gallart-Ayala H, Leitão MC, Galceran MT, Hursthouse A, Pereira CS (2011). Degradation pathway of pentachlorophenol by *Mucor plumbeus* involves phase II conjugation and oxidation-reduction reactions. J Hazard Mater.

[CR6] Chang YC, Reddy MV, Umemoto H, Sato Y, Kang MH, Yajima Y, Kikuchi S (2015) Bio-Augmentation of *Cupriavidus sp*. CY-1 into 2,4-D contaminated soil: microbial community analysis by culture dependent and independent techniques. PLoS ONE. 10.1371/journal.pone.0145057 10.1371/journal.pone.0145057 PMC4699198 26710231

[CR7] Feld L, Nielsen TK, Hansen LH, Aamand J, Albers CN (2015). Establishment of bacterial herbicide degraders in a rapid sand filter for bioremediation of phenoxypropionate - polluted groundwater. Appl Environ Microbiol.

[CR8] Ferreira - Guedes S, Mendes B, Leitão AL (2012). Degradation of 2,4-dichlorophenoxyacetic acid by a halotolerant strain of *Penicillium chrysogenum*: antibiotic production. Environ Technol.

[CR9] Gonzalez AJ, Gallego A, Gemini VL, Papalia M, Radice M, Gutking G, Planes E, Korol SE (2012). Degradation and detoxification of the herbicide 2,4-dichlorophenoxyacetic acid (2,4-D) by an indigenous *Delftia sp.* strain in batch and continuous systems. Int Biodeterior Biodegrad.

[CR10] Heggstrom MJ (2009) The sublethal effects of 2,4-D dimethylamine on wood frog tadpoles in Saskatchewan. Thesis Master of Science, University of Saskatchewan, Canada

[CR11] Hirose S, Kawahigashi H, Tagiri A, Imaishi H, Ohkawa H, Ohkawa Y (2007). Tissue-specific expression of rice CYP72A21 induced by auxins and herbicides. Plant Biotechnol Rep.

[CR12] Janicki T, Krupiński M, Długoński J (2016). Degradation and toxicity reduction of the endocrine disruptors nonylphenol, 4-tert-octylphenol and 4-cumylphenol by the non-ligninolytic fungus *Umbelopsis isabellina*. Bioresour Technol.

[CR13] Kołek T, Milecka - Tronina NA, Świzdor M, Bialońska A (2011). Hydroxylation of DHEA, androstenediol and epiandrosterone by *Mortierella isabellina* AM212. Evidence indicating that both constitutive and inducible hydroxylases catalyze 7α- as well as 7β-hydroxylations of 5-ene substrates. Org Biomol Chem.

[CR14] Kwan CY, Chu W (2004). A study of the reaction mechanisms of the degradation of 2,4-dichlorophenoxyacetic acid by oxalate-mediated photooxidation. Water Res.

[CR15] Leis M, Maufra L, Taddia L, Chicca M, Trentini P, Savorelli F (2014). A comparative toxicity study between an autochthonous *Artemia* and a non native invasive species. Ecotoxicology.

[CR16] Li K, Wu JQ, Jiang LL, Shen LZ, Li JY, He ZH, Wei P, Lv Z, He MF (2017). Developmental toxicity of 2,4-dichlorophenoxyacetic acid in zebrafish embryos. Chemosphere.

[CR17] Libralato G, Volpi Ghirardini A, Avezzù F (2010). Seawater ecotoxicity of monoethanolamine, diethanolamine and triethanolamine. J Hazard Mater.

[CR18] Mehmood Z, Williamson MP, Kelly DE, Kelly SL (1996). Human cytochrome P450 3A4 is involved in the biotransformation of the herbicide 2,4-dichlorophenoxyacetic acid. Environ Toxicol Pharmacol.

[CR19] Morshed MH, Hossain MS, Islam MAU, Ali MU, Ibrahim M, Islam MS, Islam MA (2005). Toxicity of four synthetic plant hormones IAA, NAA, 2,4-D and GA against *Artemia salina* (Leach). Int J Agric Biol.

[CR20] Nakagawa A, Osawa S, Hirata T, Yamagishi Y, Hosoda J, Horikoshi T (2006). 2,4-Dichlorophenol degradation by the soil fungus *Mortierella sp*. Biosci Biotechnol Biochem.

[CR21] Onkawa H, Inui H (2015). Metabolism of agrochemicals and related environmental chemicals based on cytochrome P450s in mammals and plants. Pest Manag Sci.

[CR22] Sarikaya R, Selvi M (2005). Investigation of acute toxicity of (2,4-dichlorophenoxy) acetic acid (2,4-D) herbicide on larvae and adult Nile tilapia (*Oreochromis niloticus L.*). Environ Toxicol Pharmacol.

[CR23] Siewiera P, Bernat P, Różalska S, Długoński J (2015). Estradiol improves tributyltin degradation by the filamentous fungus *Metarhizium robertsii*. Int Biodeterior Biodegrad.

[CR24] Słaba M, Szewczyk R, Piątek MA, Długoński J (2013). Alachlor oxidation by the filamentous fungus *Paecilomyces marquandii*. J Hazard Mater.

[CR25] Torra J, Rojano-Delgado AM, Rey-Caballero J, Royo-Esnal A, Salas ML, De Prado R (2017) Enhanced 2,4-D Metabolism in Two Resistant Papaver rhoeas Populations from Spain. Frontiers in Plant Science 8.10.3389/fpls.2017.01584 10.3389/fpls.2017.01584 PMC5602352 28955370

[CR26] Vroumsia T, Steiman R, Seigle - Murandi F, Benoit - Guyod JL (1999). Effects of culture parameters on the degradation of 2,4-dichlorophenoxyacetic acid (2,4-D) and 2,4-dichlorophenol (2,4-DCP) by selected fungi. Chemosphere.

[CR27] Vroumsia T, Steiman R, Seigle - Murandi F, Benoit - Guyod JL (2005). Fungal bioconversion of 2,4-dichlorophenoxyacetic acid (2,4-D) and 2,4-dichlorophenol (2,4DCP). Chemosphere.

[CR28] Wu X, Wang W, Liu J, Pan D, Tu X, Lv P, Wang Y, Cao H, Wang Y, Hua R (2017). Rapid biodegradation of the herbicide 2,4-dichlorophenoxyacetic acid by *Cupriavidus gilardii* T-1. J Agric Food Chem.

[CR29] Xu X, Lu Y, Zhang D, Wang Y, Zhou X, Xu H, Mei Y (2015). Toxic assessment of triclosan and triclocarbon on *Artemia salina*. Bull Environ Contam Toxicol.

[CR30] Zawadzka K, Bernat P, Felczak A, Lisowska K (2015). Carbazole hydroxylation by the filamentous fungi of the *Cunninghamella species*. Environ Sci Pollut Res.

